# Oligosaccharides isolated from *Rehmannia glutinosa* protect LPS-induced intestinal inflammation and barrier injury in mice

**DOI:** 10.3389/fnut.2023.1139006

**Published:** 2023-02-23

**Authors:** Xiao Li, Rong Gui, Xuefang Wang, Erjuan Ning, Lixian Zhang, Yi Fan, Ling Chen, Liqin Yu, Jie Zhu, Zhining Li, Lei Wei, Wei Wang, Zihong Li, Yue Wei, Xuebing Wang

**Affiliations:** ^1^Henan Natural Products Biotechnology Co., Ltd., Zhengzhou, China; ^2^Biological Center of Henan Academy of Sciences, Zhengzhou, China; ^3^College of Animal Medicine, Henan Agricultural University, Zhengzhou, China; ^4^Henan High Tech Industry Co., Ltd., Zhengzhou, China

**Keywords:** *Rehmannia glutinosa* oligosaccharide, LPS, intestinal inflammation, barrier injury, intestinal flora

## Abstract

**Objectives:**

We investigated the protective effect of *Rehmannia glutinosa* oligosaccharides (RGO) on lipopolysaccharide (LPS)-induced intestinal inflammation and barrier injury among mice.

**Methods:**

RGO is prepared from fresh *rehmannia glutinosa* by water extraction, active carbon decolorization, ion exchange resin impurity removal, macroporous adsorption resin purification, and decompression drying. LPS could establish the model for intestinal inflammation and barrier injury in mice. Three different doses of RGO were administered for three consecutive weeks. Then the weight, feces, and health status of the mice were recorded. After sacrificing the mice, their colon length and immune organ index were determined. The morphological changes of the ileum and colon were observed using Hematoxylin-eosin (H&E) staining, followed by measuring the villus length and recess depth. RT-qPCR was utilized to detect the relative mRNA expression of intestinal zonula occludens-1 *(ZO-1)* and *occludin*. The expression of inflammatory factors and oxidation markers within ileum and colon tissues and the digestive enzyme activities in the ileum contents were detected using ELISA. The content of short-chain fatty acids (SCFAs) in the colon was determined with GC. The gut microbial composition and diversity changes were determined with 16S-rRNA high-throughput sequencing. The association between intestinal microorganisms and SCFAs, occludins, digestive enzymes, inflammatory factor contents, and antioxidant indexes was also analyzed.

**Results:**

RGO significantly increased the weight, pancreatic index, thymus index, and colon length of mice compared with the model group. Moreover, it also improved the intestinal tissue structure and increased the expression of intestinal barrier-related junction proteins ZO-1 and Occludin. The contents of IL-6, IL-17, IL-1β, and TNF-α in the intestinal tissues of mice were significantly reduced. Additionally, the activities of superoxide dismutase (SOD), glutathione peroxidase (GSH-Px), and catalase (CAT) were elevated. In contrast, the malondialdehyde (MDA) content decreased. Trypsin and pancreatic lipase activities in the ileum enhanced, and the SCFA contents such as acetic acid, propionic acid, and butyric acid in the colon increased. The study on intestinal flora revealed that RGO could enhance the abundance of intestinal flora and improve the flora structure. After RGO intervention, the relative abundance of Firmicutes, *Lactobacillus*, and *Akkermania* bacteria in the intestinal tract of mice increased compared with the model group, while that of Actinomycetes decreased. The intestinal microbiota structure changed to the case, with probiotics playing a dominant role. The correlation analysis indicated that Lactobacillus and Ackermann bacteria in the intestinal tract of mice were positively associated with SCFAs, *Occludin, ZO-1*, pancreatic amylase, SOD, and CAT activities. Moreover, they were negatively correlated with inflammatory factors IL-6, IL-17, IL-1β, and TNF-α.

**Conclusions:**

RGO can decrease LPS-induced intestinal inflammation and intestinal barrier injury in mice and protect their intestinal function. RGO can ameliorate intestinal inflammation and maintain the intestinal barrier by regulating intestinal flora.

## Introduction

The incidence rate of intestinal inflammatory diseases has gradually increased in recent years, becoming a global health management problem (1). The intestinal tract is the leading site of digestion and absorption, and the intestinal mucosa has rich blood vessels. Inflammation will lead to intestinal barrier injury. Simultaneously, intestinal barrier injury is also involved in various intestinal diseases, closely associated with inflammatory bowel disease, bacterial enteritis, and Crohn's disease (2). Intestinal injury can cause emaciation, malnutrition, stunted growth, and even death of patients, among severe cases (3). The timely and appropriate application of enteral nutrition can effectively enhance the nutritional status of patients and alleviate the release of intestinal inflammatory factors. Moreover, it effectively improves intestinal mucosal injury, significantly maintaining the health of the intestinal system (4). Hormones and antibiotics for treating intestinal inflammation have noticeable therapeutic effects, but they can also cause potential damage to the body. Therefore, using natural nutritional agents without side effects for treatment and prevention is a new method for treating intestinal inflammatory diseases.

*Rehmannia glutinosa* is the root tuber of the Scrophulariaceae plant Rehmannia. It is a traditional Chinese medicine with the functions of nourishing Yin, clearing heat, tonifying blood, and stopping bleeding. Rehmannia has a long history of consumption in China. Around 1,000 years ago, in Huaiqing Prefecture and other Rehmannia-producing areas in Henan Province, people “pickled the Rehmannia into pickles, and soaked in wine and tea for consumption.” Rehmannia is still shredded and served cold or boiled into porridge. Studies have depicted that the main chemical components of *Rehmannia glutinosa* are iridoid glycosides, oligosaccharides, polysaccharides, and amino acids, which are the material basis for it to play its role (5). Oligosaccharides are low molecular weight sugar polymers formed by the condensation of 3–9 monosaccharides by glycosidic bonds (6). These oligosaccharides cannot be easily digested and hydrolyzed in the small intestine. Instead, they are utilized by probiotics after entering the hindgut to enhance the proliferation of beneficial bacteria and contribute to the stability of intestinal microecology (7). Several studies have demonstrated that intestinal microorganisms are closely related to intestinal health and function. Once the balance of intestinal flora is destroyed, it can lead to excessive consumption of the mucosal layer and accelerated apoptosis of intestinal mucosal epithelial cells, thereby damaging the intestinal mucosal barrier. Many pathogenic bacteria could invade to trigger a strong intestinal immune response, increasing the secretion of various intestinal inflammatory factors and ultimately inducing digestive and absorption dysfunction (8–10). However, it has not been reported whether RGO can ameliorate intestinal inflammation and maintain the intestinal barrier by regulating intestinal microbiota.

LPS comprises lipids and polysaccharides at the outermost layer of the cell wall of gram-negative bacteria and is an inflammatory stimulator (9). Studies have indicated that LPS can bind to TLR4 receptors on the cell surface. They activate nuclear factor-κB (NF-κB) through the MyD88 pathway and then enter the nucleus to induce the synthesis and release of cytokines. These include tumor necrosis factor-α (TNF-α), interferon-γ (IFN-γ), interleukin-6 (IL-6), etc., which causes intestinal inflammation (11). Long-term exposure to heterogeneous LPS can destroy the intestinal mucosal barrier, causing intestinal flora homeostasis (12). The amount of SCFAs produced can indirectly reflect the balance of intestinal flora, and the content of intestinal tight junction protein can also reflect the health of intestinal barrier. Therefore, in this study, LPS was used to construct an animal model for intestinal inflammation and barrier injury using mice to explore the protective effect of RGO on LPS-induced intestinal inflammation and barrier injury in mice. Moreover, the relationship between RGO and intestinal microorganisms was also assessed, thus providing the theoretical basis for applying RGO in intestinal inflammatory diseases.

## Materials and methods

### Materials and reagents

Fresh Rhizomes of *Rehmannia glutinosa* were obtained from Wuzhi County, Henan Province, China (35° 1′23″ north latitude, 113° 18′76″ east longitude) in December 2021. Stachyose (NO. 112031-201701), sucrose (NO.1 11507-202105), raffinose (NO. 190225-201901), and verbascose (NO. 111530-201914) were purchased from China Institute for Food and Drug Control.

Dexamethasone tablets were purchased from Tangshan Longkang Pharmaceutical Co., Ltd.

The lipopolysaccharide (LPS) was purchased from Sigma Company in the United States. The detection kits for TNF-α, IL-6, IL-17, and IL-1β were purchased from Shanghai Enzyme-linked Biotechnology Co., Ltd. Moreover, the detection kits for SOD, MDA, GSH-Px, and CAT were purchased from the Nanjing Jiancheng Bioengineering Research Institute. Additionally, the digestion enzyme test kits were purchased from Beijing SOLEBAR Technology Co., Ltd. All other chemicals, solvents, and reagents were of pure analytical grade.

### Preparation of RGO

Fresh Rhizomes of *Rehmannia glutinosa* were cut into small pieces of 5–10 mm after being washed, added four times the amount of water, and extracted twice at 90°C, at 1 h duration. The two extracts were combined, adding activated carbon (2 g/100 ml) and activated clay (2 g/100 ml) to the extract. It was stirred and decolored at 80°C for 30 min, then centrifuged. The supernatant was passed through 001 × 7 cation exchange resin column (diameter: high = 6:1), D201 type anion exchange resin column (diameter: high = 6:1), D101 macroporous adsorption resin column (diameter: high = 10:1) one by one, sample volume (mL): resin column volume = 1:1.5, flow rate was 500 mL/h. Finally, the macroporous adsorption resin effluent was collected and concentrated and dried at 60°C to get white powder, that is RGO.

The type and content of oligosaccharides in RGO were detected using high-performance liquid chromatography (HPLC) (Agilent1260), configured using a Refractive Index Detector (RID) (13). The standard reference substances of sucrose, stachyose, raffinose, and mulberry sugar were weighed precisely and prepared with 70% acetonitrile aqueous solution into the standard reference solution with a concentration of 0.5 mg/mL, respectively. The RGO powder was also weighed precisely, and prepared with 70% acetonitrile aqueous solution into the sample solution with a concentration of 1 mg/mL. The chromatographic column was Agilent ZORBOX NH2 (4.6 mm × 250 mm, 5 μm); the mobile phase was acetonitrile: water (7:3); the injection volume was 10 μL, the flow rate was 1.0 mL/min, and the temperature of column incubator was 40°C. The temperature of the detection was 50°C with RID. The types of oligosaccharides in RGO were determined by comparing the HPLC peaks of reference substance with those in RGO, and the content of oligosaccharides was calculated by external standard method.

### Animal and experimental design

We purchased 36 KM mice from Henan Scribes Biotechnology Co., Ltd. The mice were fed for 7 days before the experiment to acclimate to the environment. The mice were randomly divided into six groups, namely normal (N), model (M), treatment (T), RGO low dose (RL, 0.25 g/kg), RGO medium dose (RM, 0.5 g/kg), and RGO high dose (RH, 1 g/kg) groups. Six mice were in each group, and the test period was 21 days. Except for the normal group, on the 9th, 13th, 17th, and 21st days, 0.2 mL of normal saline was injected intraperitoneally. 0.2 mL of 1 mg/kg LPS was injected intraperitoneally in all other groups to develop mice models of intestinal inflammation and barrier injury (14, 15). From the first day of the test, mice in RL, RM, and RH groups were provided 0.2 mL of RGO solution by gavage once a day for 21 days. The mice in the N, M, and T groups were gavaged with an equal volume of normal saline daily. After each intraperitoneal LPS injection, the mice in the T group were gavaged with 0.2 mL dexamethasone solution at 0.5 mg/kg dose 30 min. All the groups were fed adequate food and free to drink water. The weight of mice was determined on the 1^st^, 7^th^, 14^th^, and 21^st^ days of the test. Additionally, the food intake, fecal properties, and health status of mice were observed and recorded daily.

### Sample collection

On the 21^st^ day of the test, the mice were sacrificed under ether anesthesia after 6 h of LPS treatment and dissected. The thymus, spleen, and liver tissues were collected, rinsed with PBS solution, blotted dry using filter paper, and weighed. Then the immune organ index of the mice was calculated. The whole colon was taken, and the length of the colon was measured. The ileum and colon tissues and their contents were collected to detect related indicators. A part of the intestinal tissue was placed in 4% paraformaldehyde for histopathological analysis. Then, the rest of the intestinal tissue and contents were stored at −80°C.

Immune organ index = weight of organ (mg)/body weight (g).

### Histopathology

The ileum and colon tissues of mice were extracted from a 4% paraformaldehyde solution. About 1 cm of the middle segment was dehydrated using ethanol, hyalinized in xylene, embedded in paraffin, and sectioned (4–6 μm). The pathologic slices were made with Hematoxylin-eosin (H&E) staining, and the morphology of intestinal tissues was observed using a light microscope (Olympus, DP-72, Tokyo, Japan). The jejunum villus length, the colonic fold height, and the depth of the crypt of the ileum and colon were evaluated.

### RT-qPCR detection

The total RNA of the ileum and colon mucosa was extracted using the Trizol method. Then, 1,000 ng of total RNA was reverse transcribed into cDNA with RT Super Mix and stored at −80°C. β-actin was utilized as the reference gene. The primer sequences of the target gene and the reference gene are shown in Table 1. The cDNA was used as the template for real-time PCR (16). Reaction procedures: High-temperature denaturation was performed at 95°C for 10 min, renaturation at 95°C for 15 s, and primer extension at 60°C for the 60 s. This was repeated for 40 cycles. Cycle threshold (Ct) values were utilized for the relative quantification of RT-qPCR amplification. The Ct value method compared the expression of target genes associated with β-actin.

**Table 1:** Primer sequence.

**Gene name**	**Upstream primer**	**Downstream primer**
ZO-1	5′-GGGTCATCATCTCTGCACCT-3′	5′-GGTCATAAGTCCCTCCACGA-3′
Occludin	5′-AACAACCCCTTCCAAGTTCC-3′	5′-CTCCCAGAGTTCCGATTCAC-3′
β-actin	5′-ACCTCCAGGACGACGACTTTGAT-3′	5′-GTGTCTTCTGCACGTACTCCA-3′

### Enzyme-linked immunosorbent assay

Precooled normal saline was added to the ileum and colon tissues of mice at the ratio of weight (g): volume (ml) = 1:10, respectively. The homogenate was mechanically homogenized at 3,000 r/min and centrifuged for 15 min under ice-water bath conditions. The supernatant was taken, and according to the kit's instructions, the contents of IL-6, IL-17, TNF-α, IL-1β, MDA, SOD, GSH-Px, and CAT were determined.

### Digestive enzyme detection

Precooled normal saline was added to the middle ileum of mice at the ratio of weight (g): volume (ml) = 1:10. A high-speed grinder centrifuged them at 3,000 r/min for 15 min, and the supernatant was taken. The activities of trypsin, lipase and amylase were determined based on the kit's instructions.

### SCFA content in feces

The colon contents of mice were weighed accurately. Methanol was added at a ratio of 1:5 (mg: μL). The mixture was stirred for 30 s to form a uniform suspension. A small amount of concentrated sulfuric acid solution was added to adjust the pH to 2–3. The samples were left at room temperature for 10 min through continuous shaking. Then, the samples were centrifuged at 12,000 rpm for 10 min. Ten microliter of supernatant was taken, and the content of SCFA was determined through the Shimadzu GC-2014C gas chromatograph (Shimadzu, Japan), the flame ionization detector, and the DB-FFAP capillary column (30 m × 0.25 m × 0.25 mm) (17).

### 16S rRNA high-throughput sequencing analysis of intestinal flora

The colonic contents of mice were obtained through 16S rRNA high-throughput sequencing, commissioned by Shanghai Parsono Biotechnology Co., Ltd.

### Statistical analysis

Microsoft Excel software were used for Preliminary statistical. GraphPad Prism 8 software was used for image processing. All data were collected in triplicate and the average value was used for analysis and the data were statistically compared for significant differences by one-way analysis of variance (One-Way ANOVA) and Duncan's multiple comparisons using SPSS 22.0 software.

## Results

### Identification and content determination of RGO

**Figure 1:**
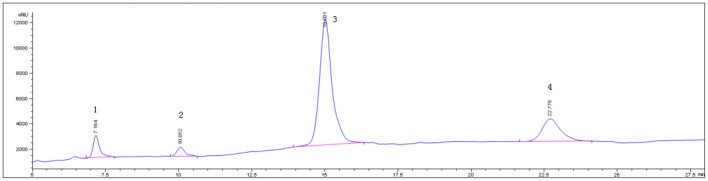
RGO HPLC chromatogram (1 sucrose, 2 raffinose, 3 stachyose, 4 verbascose).

Determination of main components of RGO by HPLC, The peak positions of sucrose, raffinose, stachyose and verbascose were 7.164, 10.052, 15.001, 22.778 min, respectively, The RGO isolated in this experiment have chromatographic peaks in the corresponding position. The contents of sucrose, raffinose, stachyose, and verbascose in the prepared RGO were 7.52, 5.19, 81.02, and 4.85%, respectively, Moreover, the total amount of oligosaccharides was 91.06%. The HPLC chromatogram is shown in Figure 1.

### Effects of RGO on body weight, organ index, and colon length in LPS mice

**Figure 2:**
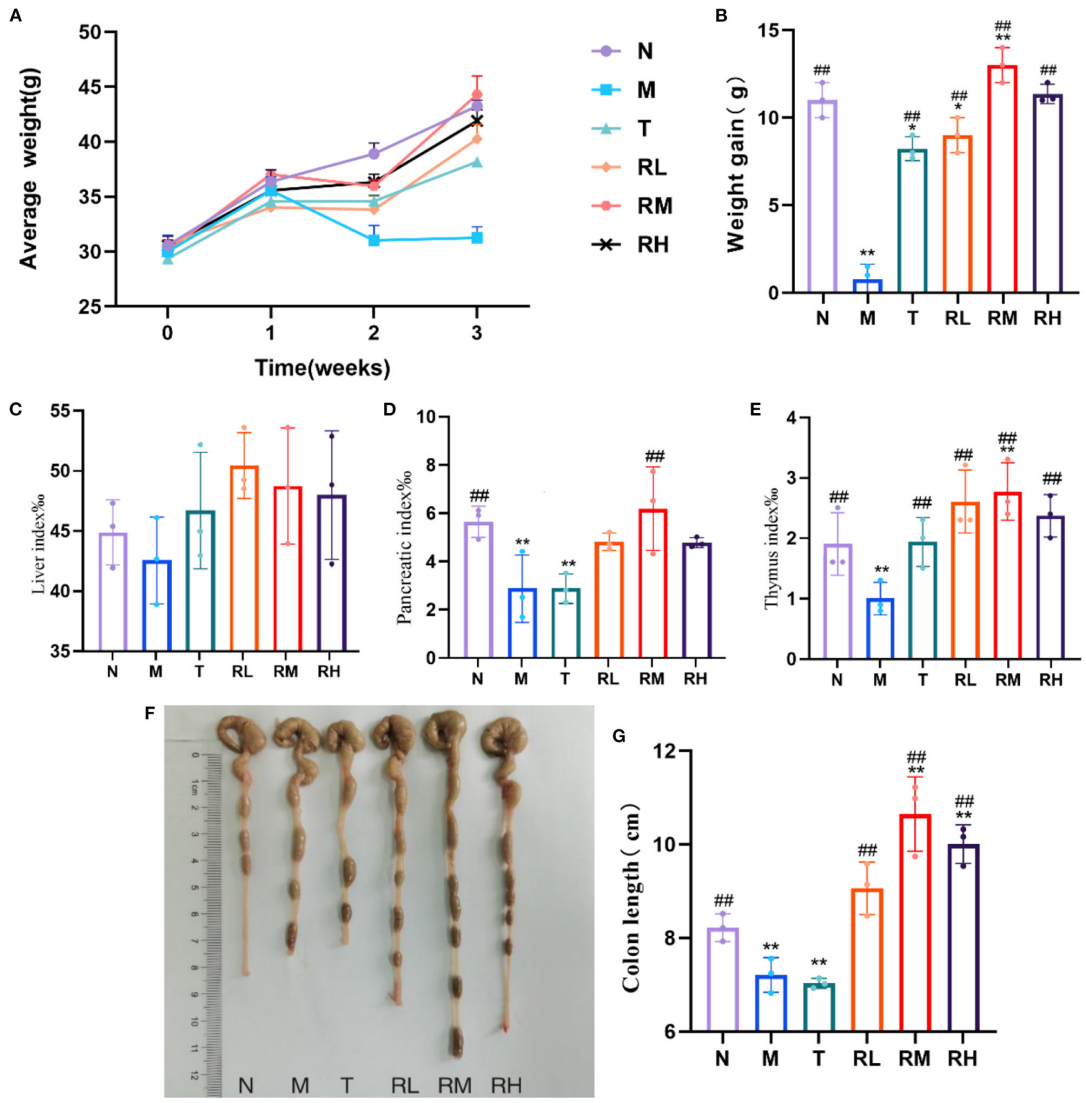
Changes of body weight, immune organ index and colon length in mice during the test. **(A)** Weekly body weight of mice. **(B)** Body weight gain of mice. **(C)** Liver index. **(D)** Pancreatic index. **(E)** Thymus index. **(F)** Colon of mice. **(G)** Length of colon. Compared with group N: *P < 0.05, **P < 0.01; compared with group M. ^#^*P* < 0.05, ^##^*P* < 0.01.

As shown in Figure 1, no significant difference was observed in the body mass of mice from each group before the intraperitoneal LPS injection. After the LPS injection, the mice in the M group showed symptoms of movement retardation, in appetence, soft stools, tears, listlessness, and messy fur. During the experiment, the body weight gain of mice in the M group was significantly decreased (*P* < 0.01) compared with the N group. Moreover, the body weight gain of mice in RL, RM, RH, and T groups was significantly increased compared with the M group (*P* < 0.01). Among them, the weight gain in the RL and T groups was significantly lower than in the N group (*P* < 0.05). The body weight gain in the RH group revealed no significant difference from that in the N group (*P* < 0.05). The body weight gain in the RM group was significantly higher than the N group (*P* < 0.05) (Figures 2A, B). Thus, RGO could inhibit LPS-induced weight loss within mice.

The liver index of mice in each experimental group had no significant difference compared with group N. In contrast, the pancreas and thymus indexes of mice in the M and T groups were significantly reduced (*P* < 0.01). Compared with the M group, the pancreas index of mice in the RM group was significantly elevated (*P* < 0.01). Moreover, the thymus index of mice in RL, RM, RH, and T groups was significantly increased (*P* < 0.01) (Figures 2C–E). We also determined the colon length of mice within each experimental group. Compared with the N group, the colon length of mice in the M and T groups was significantly reduced (*P* < 0.01). However, the colon length of mice in RL, RM, and RH groups increased significantly compared with the M group (*P* < 0.01) (Figures 2F, G). These results demonstrated that LPS induced atrophy of the pancreas, thymus, and colon in mice. RGO intervention could decrease the atrophy of the pancreas, thymus, and colon in LPS mice and protect them.

### Effects of RGO on intestinal epithelial barrier in LPS mice

**Figure 3:**
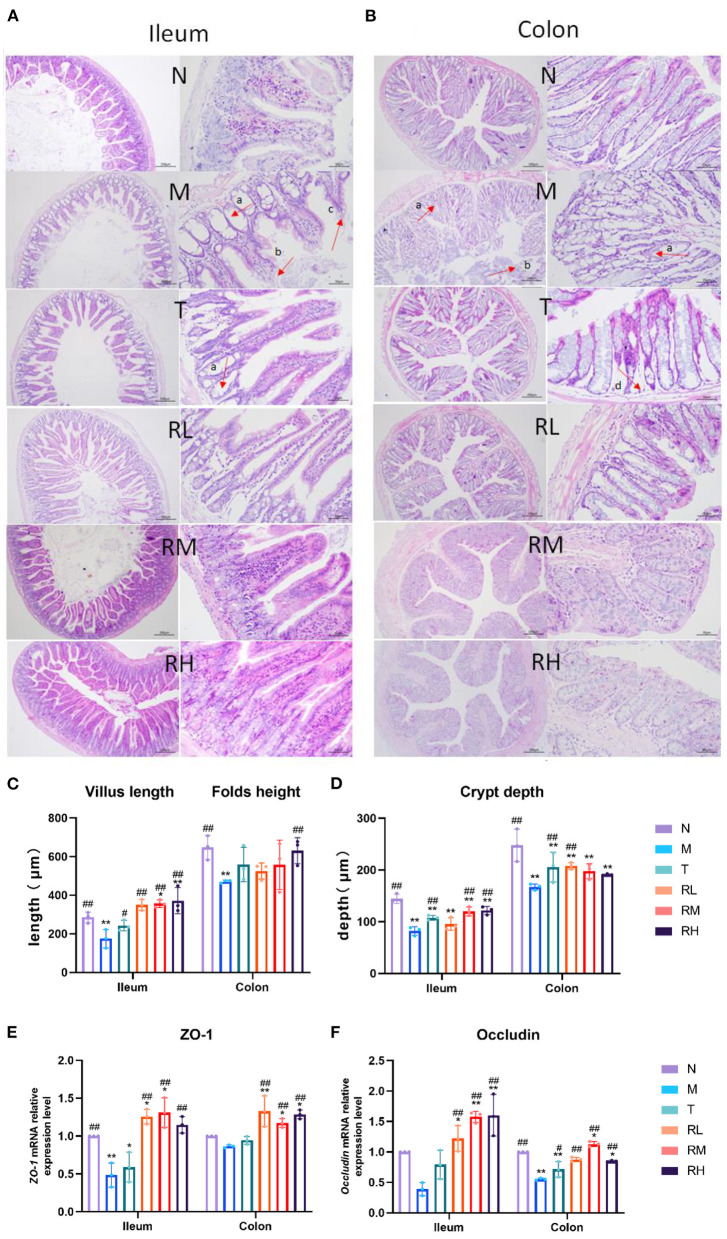
Effect of RGO on intestinal epithelial barrier in LPS mice. **(A)** Pathological assessment of H&E-stained mice ileum sections; magnification 10× and 40×. **(B)** Pathological assessment of H&E-stained mice colon sections; magnification 10× and 40×. **(C)** Villus length and folds height. **(D)** Crypt depth. **(E)** *ZO-1* mRNA relative expression level. **(F)** *Occludin* mRNA relative expression level (a. Intestinal gland necrosis; b. Epithelial cell exfoliation; c. The villus becomes shorter; d. Necrosis of intestinal gland basal layer). Compared with group N: ^*^P < 0.05, ^**^P < 0.01; compared with group M. ^#^*P* < 0.05, ^##^*P* < 0.01.

We used H&E-stained to detect the pathological changes in the ileum and colon and investigate the effect of RGO on the intestinal epithelial barrier of LPS mice (Figures 3A, B). From the pathological section of the ileum and colon, it could be observed that the intestinal mucosa of mice in the N group was intact. Moreover, the villi of the small intestine were arranged closely and regularly, and the morphology of epithelial cells was normal. In the M group, intestinal gland necrosis, villus shortening, necrosis, and abscission of part of villous epithelium in the ileum, intestinal gland necrosis, goblet cell abscission, and other pathological conditions in the colon were observed. In each administration group, the exfoliation of intestinal epithelial cells was milder, the villus was arranged orderly, inflammatory cell infiltration was reduced, and infection symptoms were alleviated. The villus length and crypt depth of the ileum and the height and crypt depth of colonic folds were evaluated (Figures 3C, D). Compared with group N, the ileal villi length of mice in group M was significantly shortened (*P* < 0.01). Additionally, the height of colonic folds was significantly reduced (*P* < 0.01), and the depth of the ileal colonic recess was significantly enhanced (*P* < 0.01). This indicated that LPS caused intestinal villi damage, recess deepening, and intestinal epithelial barrier injury among mice. The villus length of the ileum of the mice in the RM and RH groups increased significantly (*P* < 0.01) compared with the M group. Moreover, the height of colonic folds in the RH group increased significantly (*P* < 0.01), and the depth of the ileal crypt in the RL, RM, and RH groups significantly decreased (*P* < 0.01).

RT-qPCR detected the relative mRNA expression of the intestinal barrier-related junction proteins *Occludin* and *ZO-1* (Figures 3E, F). The results indicated that the relative mRNA expression of *Occludin* and ZO-1 in mice ileum and colon mucosa in group M was lower than in group N. The relative mRNA expression of *Occludin* and *ZO-1* in mice ileum and colon mucosa in RL, RM, and RH groups was significantly increased compared with the M group (*P* < 0.01). These results indicated that RGO could enhance the intestinal morphology of LPS mice, increase the length of the intestinal villus, the height of the colonic fold, and the depth of the ileocolic crypt. Furthermore, it can increase the relative mRNA expression of intestinal tight junction protein and alleviate intestinal morphology and epithelial barrier damage in LPS mice.

### Effects of RGO on intestinal inflammation and oxidative indexes in LPS mice

**Figure 4:**
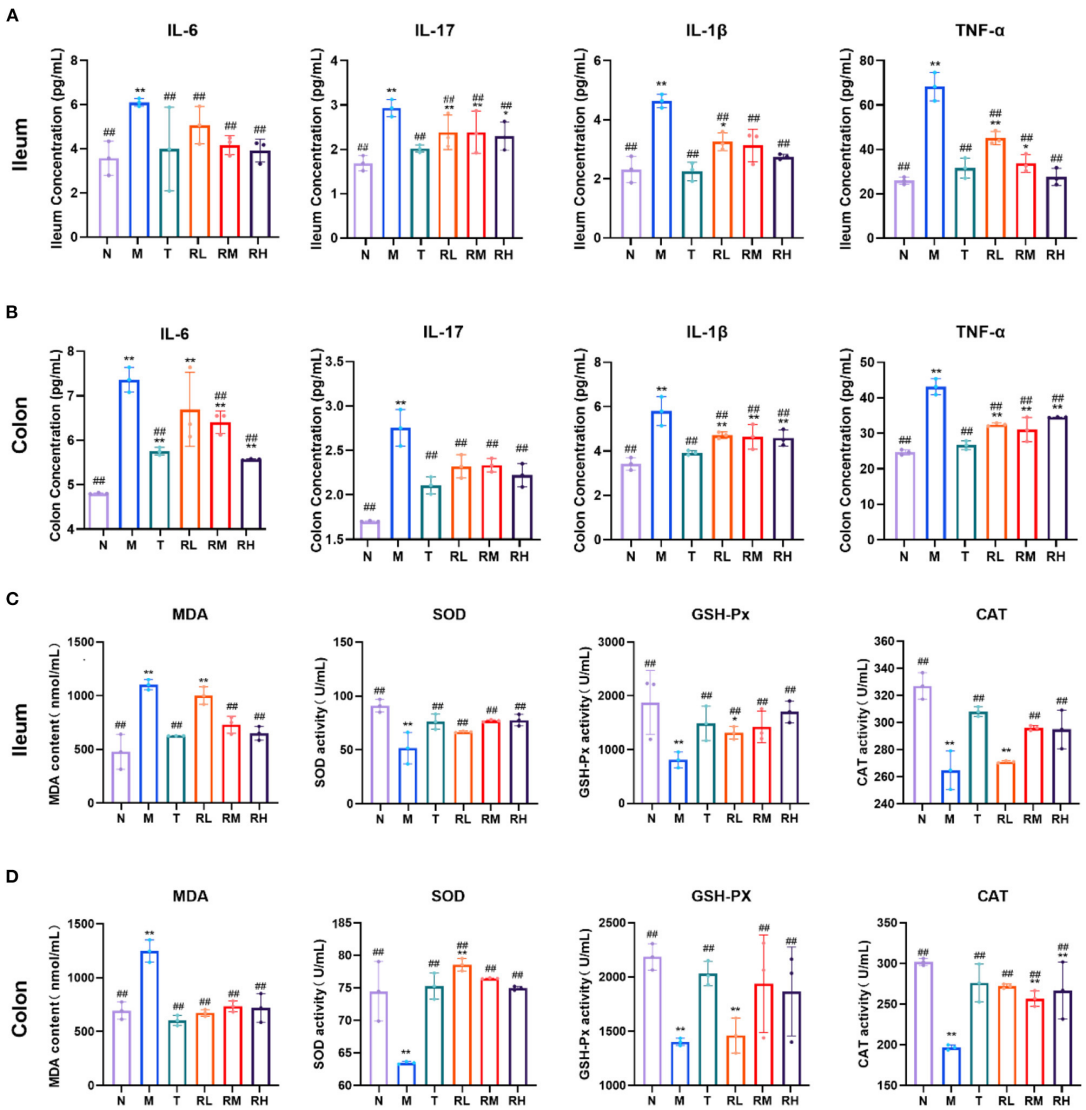
The effect of RGO on intestinal inflammatory factors and oxidative indexes in LPS mice. **(A)** IL-6, IL-17, IL-1β, and TNF-α levels in the ilenm. **(B)** IL-6, IL-17, IL-1β, and TNF-α levels in the colon. **(C)** MDA, SOD, GSH-Px, and CAT levels in the ilenm. **(D)** MDA, SOD, GSH-Px, and CAT levels in the colon. Compared with group N: *P < 0.05, **P < 0.01; compared with group M. ^#^*P* < 0.05, ^##^*P* < 0.01.

ELISA could detect the expression of related inflammatory factors in the ileum and colon and identify the effect of RGO on intestinal inflammation in LPS mice (Figures 4A, B). The results indicated that compared with the N group, the levels of IL-6, IL-17, IL-1β, and TNF-α in the ileum and colon tissues of the M group elevated significantly (*P* < 0.01). Compared with the M group, the levels of IL-6, IL-17, IL-1β, and TNF-α in the ileum of mice in RL, RM, and RH groups were significantly decreased (*P* < 0.01). However, there was no significant difference in the level of IL-6 in the colon between the RL and M groups. IL-6, IL-17, IL-1β, and TNF-α levels in the colon of RL, RM, and RH groups were significantly reduced (*P* < 0.01). These results depicted that RGO intervention could decrease the levels of intestinal inflammatory factors in LPS mice.

Inflammatory injury is often accompanied by oxidative damage to LPS-induced intestinal mucosal injury. Therefore, intestinal oxidative indicators were also measured in LPS mice. The results indicated that in the ileum tissue of mice, compared with group N, the MDA level in group M increased significantly (*P* < 0.01). In contrast, the SOD, GSH-Px, and CAT levels decreased significantly (*P* < 0.01). Compared with the M group, the MDA levels in RL, RM, RH, and T groups decreased significantly (*P* < 0.01). However, the SOD and GSH-Px levels increased significantly (*P* < 0.01). CAT levels in RL, RM and T groups also increased significantly (*P* < 0.01) (Figure 4C). In the colon tissue of mice, the level of MDA in group M was significantly elevated compared with group N (*P* < 0.01). In contrast, the SOD, GSH-Px, and CAT levels were significantly decreased (*P* < 0.01). After RGO intervention, the SOD, GSH-Px, and CAT levels in T, RM, and RH groups were significantly increased compared with group M (*P* < 0.01). The SOD and CAT levels in group RL were also significantly enhanced (*P* < 0.01), but the GSH-Px levels were insignificant (Figure 4D). These results indicated that RGO intervention could improve the antioxidant capacity of the intestinal tract of mice.

### Effects of RGO on intestinal digestive enzymes in LPS mice

**Figure 5:**
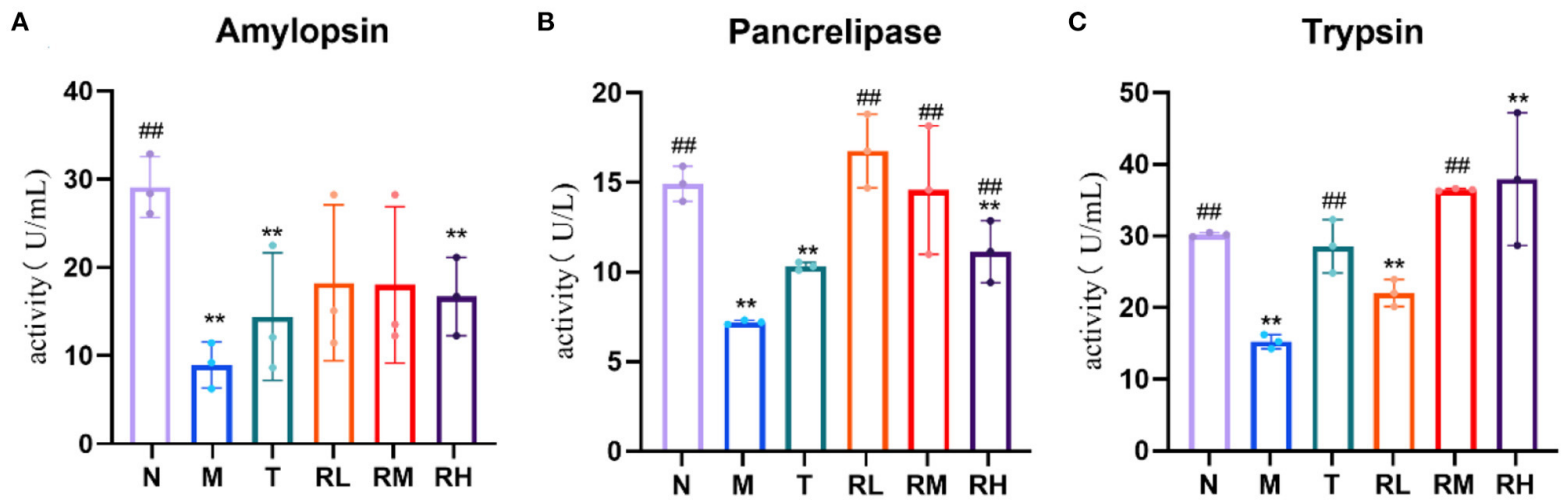
**(A–C)** Effect of RGO on digestive enzyme activity in mice. Compared with group N: *P < 0.05, **P < 0.01; compared with group M. ^#^*P* < 0.05, ^##^*P* < 0.01.

Compared with the N group, the pancreatic amylase, lipase, and trypsin activities in the ileum of mice in the M group were significantly decreased (*P* < 0.01). Compared with the M group, the activities of pancreatic lipase in RL, RM, and RH groups were significantly enhanced (*P* < 0.01). Trypsin activity in group RM was significantly increased (*P* < 0.01). However, there was no significant difference in pancreatic amylase activity (Figure 5). These results indicated that RGO intervention could restore the activities of trypsin and lipase within the intestinal tract of LPS mice.

### Effects of RGO on the content of intestinal SCFAs in LPS mice

**Figure 6:**
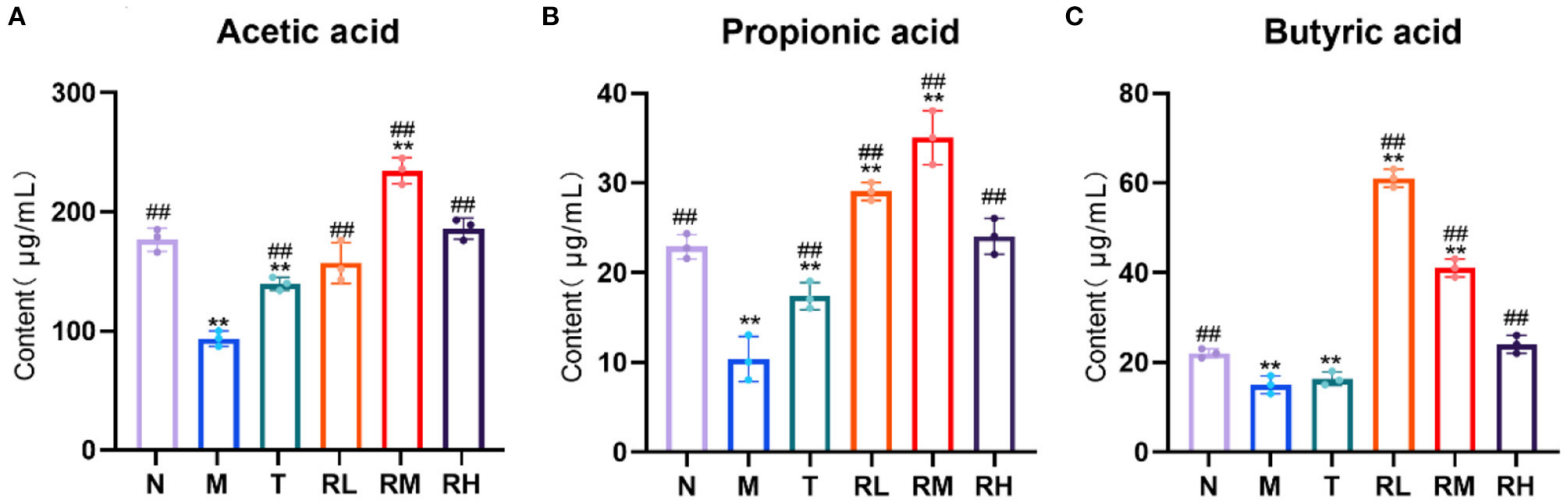
**(A–C)** Effect of RGO on SCFAs in mice. Compared with group N: *P < 0.05, **P < 0.01; compared with group M. ^#^*P* < 0.05, ^##^*P* < 0.01.

SCFAs are metabolites of the intestinal flora. Compared with the N group, acetic acid, propionic acid, and butyric acid in the colon contents of the mice in the M group were significantly decreased (*P* < 0.01). It depicted that LPS had an inhibitory effect on the production of SCFAs. Compared with the M group, acetic acid, propionic acid, and butyric acid in RL, RM, and RH groups were significantly elevated (*P* < 0.01). Among them, the contents of acetic acid and propionic acid in the RM group were the highest, while the butyric acid in the RL group was the highest (Figure 6). These results indicated that RGO intervention could enhance the content of SCFAs in the intestinal tract of LPS mice. Moreover, we also suggested that RGO could elevate the content of SCFAs by controlling the composition of the intestinal microorganisms.

### Effects of RGO on intestinal flora of LPS mice

**Figure 7:**
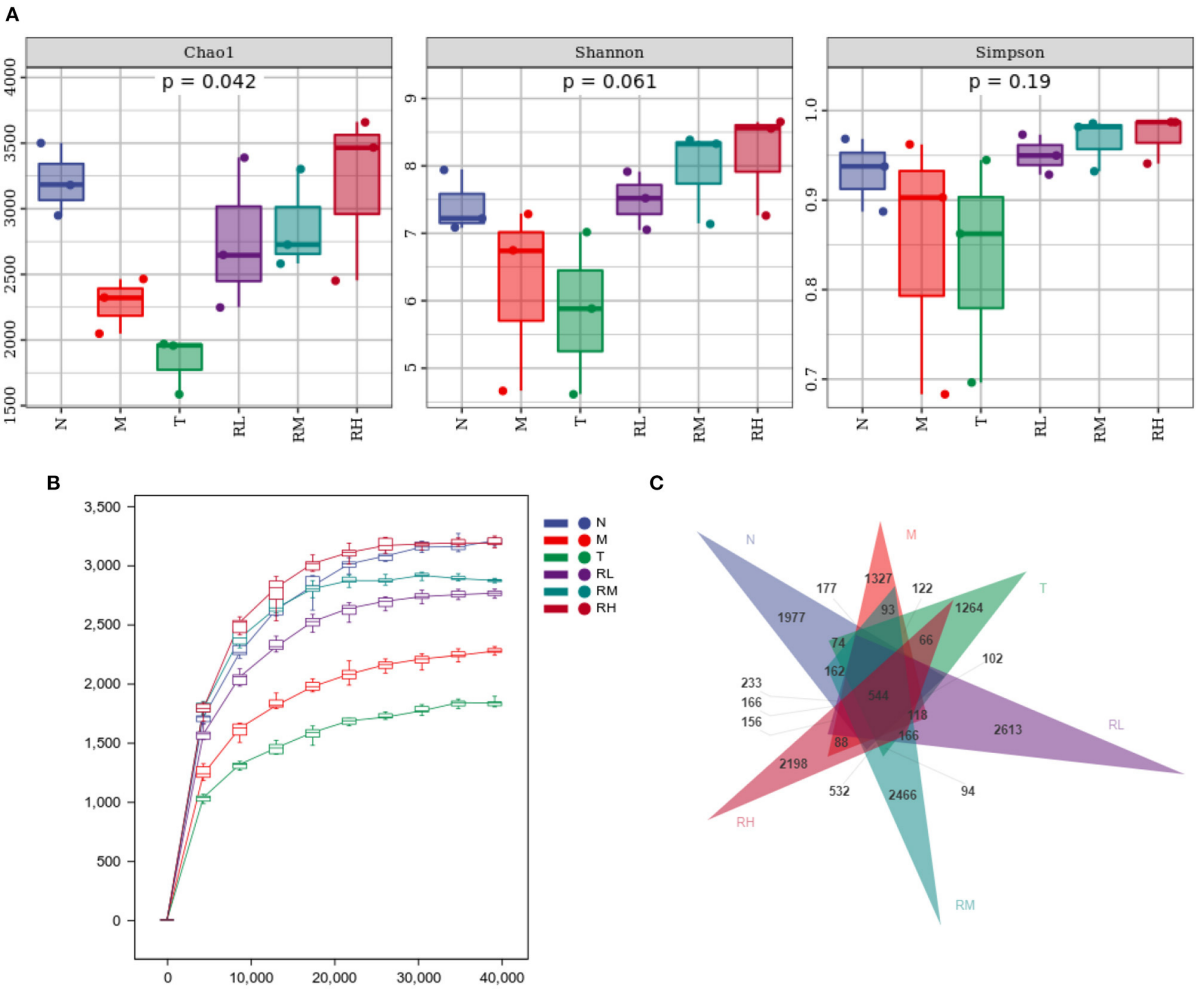
Effect of RGO on intestinal flora diversity in mice. **(A)** Alpha diversity index. **(B)** Dilution curve. **(C)** OTUs exclusivity analysis.

16S rRNA could determine the diversity and species richness of intestinal microorganisms in mice from different treatment groups. Alpha diversity analysis revealed that the species richness index Chao1 ofN, RL, RM, and RH groups was much higher than that of M and T groups, showing significant differences (*P* < 0.05). At the same time, the Shannon diversity index and Simpson diversity index were also higher than the M and T groups, without any significant difference (Figure 7A). The Rarefaction curves revealed that all curves tended to be parallel as the number of sequences increased, depicting that the sequencing data met the analysis needs (Figure 7B). Exclusivity analysis of OTUs showed that 14,738 OTUs were obtained using alignment. The proportions of annotated OTUs at the phyla, genus, and species levels were 98.14, 40.73, and 5.68%, respectively. The number of bacterial species in the RL, RM, RH, and N groups was significantly higher than in M and T groups (*P* < 0.05). There was no significant difference between the M and T groups. Compared with the M group, the number of species in RL, RM, and RH groups elevated by 14.55% (*P* < 0.05), 19.09% (*P* < 0.05), and 22.74% (*P* < 0.05), respectively (Figure 7C).

**Figure 8:**
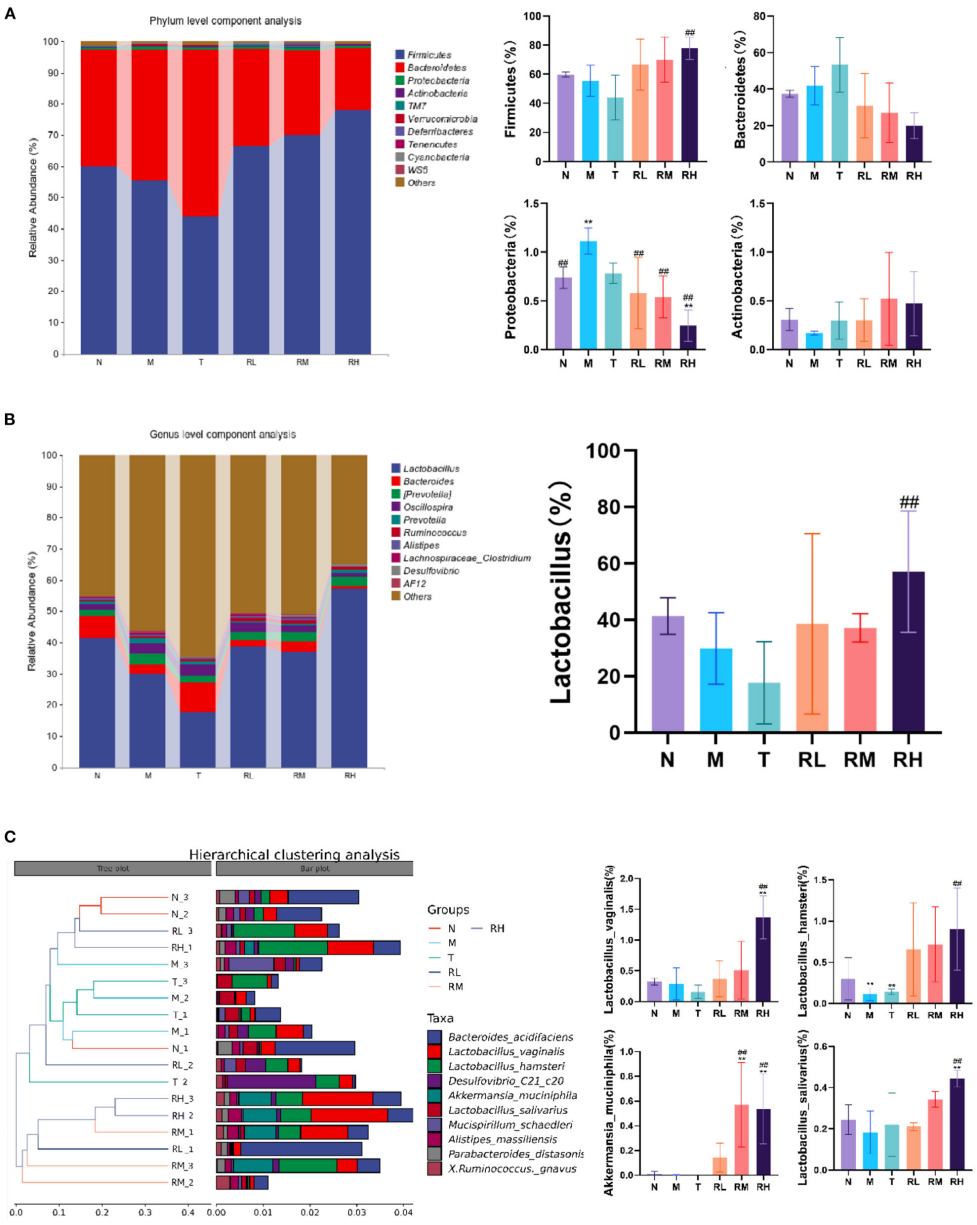
Effect of RGO on intestinal microflora structure in mice. **(A)** Phylum level composition analysis. **(B)** Genus level composition analysis. **(C)** Species level cluster analysis. Compared with group N: *P < 0.05, **P < 0.01; compared with group M. ^#^*P* < 0.05, ^##^*P* < 0.01.

At the phyla level, the microflora with a larger abundance of mouse intestinal microbes was mainly *Firmicutes, Bacteroides, Proteobacteria*, and *Actinobacteria*. *Firmicutes* and *Bacteroides* had the highest relative abundance, having more than 90% of the total microbial biomass (Figure 8A). Compared with group N, the relative abundance of *Firmicutes* among intestinal microorganisms of mice in group M was significantly decreased. The relative abundance of *Proteobacteria* was significantly enhanced (*P* < 0.01). The abnormal expansion of *Proteobacteria* reduced the ability to regulate the balance of the intestinal microbial community. The increase in *Proteobacteria* was considered a potential feature of ecological imbalance and disease risk (18). After RGO intervention, compared with the M group, the relative abundance of *Proteobacteria* in RL, RM, and RH groups decreased significantly (*P* < 0.01), while the relative abundance of *Firmicutes* increased. The relative abundance of *Firmicutes* in the RH group increased significantly (*P* < 0.01).

At the genus level, Lactobacillus and *Bacteroides* were the primary genera of intestinal microorganisms in mice (Figure 8B). The relative abundance of the two genera in group N was 41.40 and 7.10%, respectively. Compared with group N, the relative abundance of Lactobacillus in the M and T groups was reduced to 29.87 and 17.68%, respectively. Lactic acid bacteria have been a common probiotic to regulate intestinal ecological balance. After RGO intervention, the relative abundance of Lactobacillus in the RH group (57.12%) was significantly higher than that in the M group (*P* < 0.01). In contrast, the relative abundance of *Bacteroides, Prevotella*, and *Oscillospira* had no significant change.

At the species level, intestinal microorganisms among mice with relatively high abundance included *Bacteroides barnesiae, Lactobacillus vaginalis, Lactobacillus hamsteri, Akkermania musciniphila*, and *Lactobacillus salivarius*. Compared with the M group, the relative abundance of *Akkermania musciniphila* in the RM and RH groups was significantly enhanced (*P* < 0.01). Moreover, the relative abundance of *Lactobacillus vaginalis, Lactobacillus hamster*, and *Lactobacillus salivarius* in the RH group significantly increased (*P* < 0.01). Cluster analysis revealed that the intestinal flora structure of the *Rehmannia glutinosa* oligosaccharides group was clustered into one group. Thus, the effects of different doses of RGO on intestinal microorganisms were consistent (Figure 8C). Therefore, RGO regulates intestinal flora imbalance in LPS mice.

### Correlation between intestinal microorganisms and SCFAs, tight junction proteins, digestive enzymes, inflammatory factors, and antioxidant indexes

**Figure 9:**
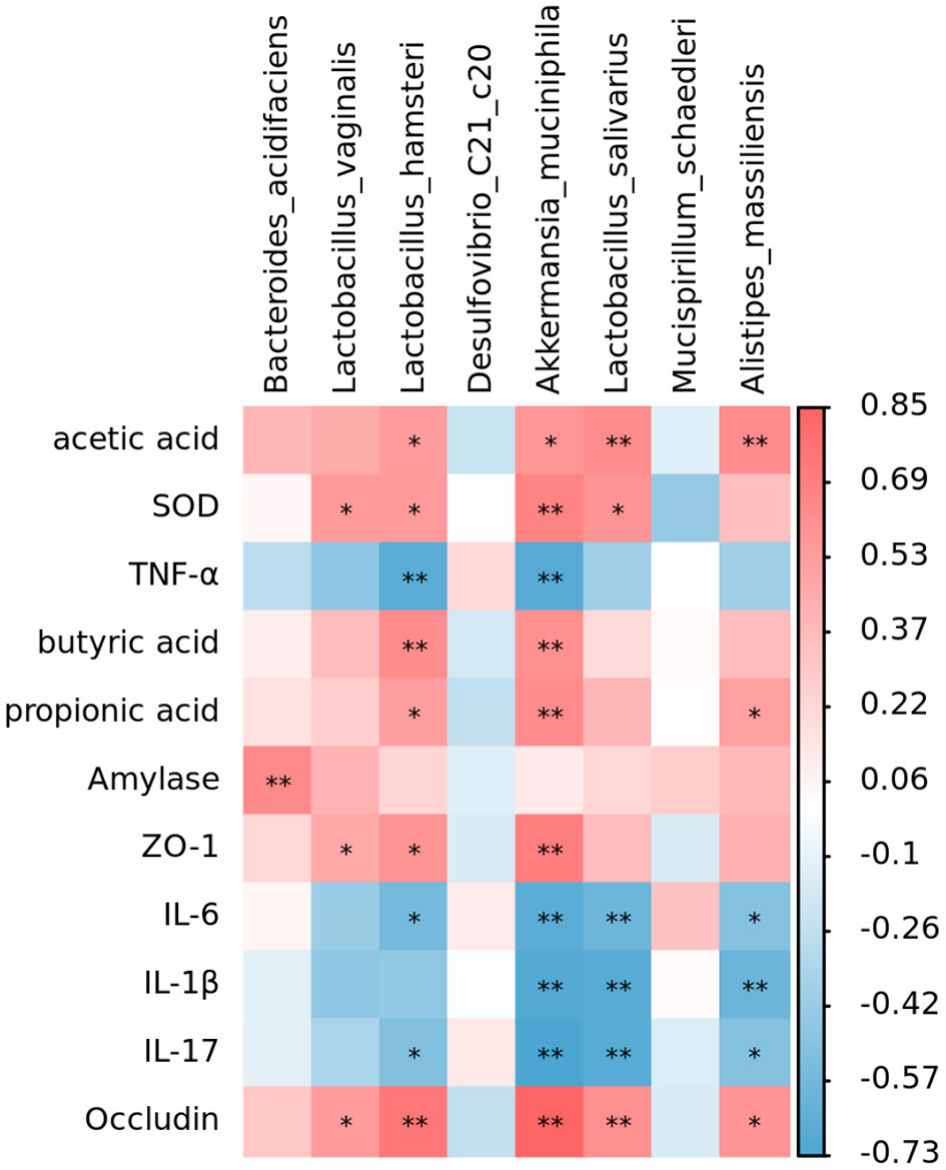
Correlation analysis of intestinal flora. *p < 0.05, **p < 0.01.

The analysis revealed that *Lactobacillus, Akkermansia*, and *Alistipes massiliensis* in the intestinal tract of mice were positively associated with SCFAs, *Occludin, ZO-1*, pancreatic amylase, and SOD activities (Figure 9). Moreover, they were negatively correlated with inflammatory factors. Such as IL-6, IL-17, IL-1β, and TNF-α. However, *Mucispirillum schaedleri* and *Desulfovibrio* have been negatively associated with SCFAs, tight junction associated proteins, digestive enzymes, and SOD activities and positively correlated with inflammatory factors. RGO intervention significantly elevated the relative abundance of *Lactobacillus, Lactobacillus*, and *Akkermansia*, combined with the structural changes of intestinal flora. Therefore, we speculate that RGO may be by increasing the proportion of beneficial bacteria in the intestine to enhance SCFA production, enhance the expression of intestinal tight junction proteins, inhibit the release of inflammatory factors, and elevate antioxidant activity. Moreover, RGO alleviated intestinal inflammation and barrier injury due to LPS. The mechanism of RGO relieving intestinal inflammation in mice needs to be further analyzed.

## Discussion

The difficulty in preventing intestinal inflammation and the strong recurrence are the problems in treating this disease (19). Therefore, human health must appropriately apply natural and non-irritating bioactive substances to enhance intestinal inflammation and maintain intestinal barrier function (1). *Rehmannia glutinosa* oligosaccharide, a natural bioactive substance, can proliferate probiotics and improve immunity (6), with high application value. Therefore, this study explored the effects of RGO on LPS-induced intestinal inflammation and barrier injury among mice.

LPS exists in the outer membrane of Gram-negative bacteria, stimulating macrophages to secrete proinflammatory cytokines, including IL-6, IL-1β, and TFN-α, while promoting the synthesis and release of inflammatory cytokines (20). The excessive secretion of IL-6, IL-17, IL-1β, and TNF-α has a crucial role in the pathogenesis of intestinal inflammation (21). TNF-α can enhance intestinal permeability by regulating the integrity of intestinal epithelial cells (22, 23). Therefore, blocking the secretion of these cytokines can be an effective strategy for treating intestinal inflammation. This study revealed that RGO could reduce the excessive secretion of inflammatory factors IL-6, IL-17 and IL-1β, and TNF-α within the intestine due to LPS.

Studies have indicated that the body has a strong oxidative stress response, and the antioxidant capacity of cells will be reduced when intestinal inflammation occurs. Excessive free radicals will act on lipid peroxidation, producing a large amount of MDA, thereby damaging the structure and function of proteins (12). SOD, GSH-Px, and CAT are the most important antioxidant enzymes in the body. Their main functions are to eliminate free radicals and reactive oxygen species, thus preventing peroxide production (24). RGO can alleviate the oxidative stress due to LPS, specifically manifested by enhancing the activities of SOD, GSH-Px, and CAT and inhibiting the enhancement of MDA levels.

The villus height and crypt depth could reflect the digestion and absorption ability of the small intestine. The higher the villus, the larger the number of peripheral intestinal epithelial cells. Moreover, the larger the contact area between the intestine and nutrients, the stronger the digestion and absorption of nutrients. The depth of the crypt becomes shallow, depicting that the maturation rate of intestinal epithelial tissue increases (25). Additionally, the ability to secrete digestive fluid is more substantial, and the intestinal absorption capacity is stronger. The plica is rich in glands and lymphoid tissue; the higher the plicae, the more excellent intestinal transport, and absorption capacity. Therefore, the higher the villi and plicae, the shallower the crypts and has better the capacity of the intestinal tract to digest and absorb nutrients. This experiment showed that the intervention of RGO could enhance the length of the ileal villi and the height of the colonic plicae. Moreover, it can reduce the depth of crypts in LPS mice, suggesting that RGO could restore the structural damage from the LPS-induced intestinal inflammation among mice.

*Occludin* and *ZO-1* are essential tight junction proteins of the intestinal barrier structure (26). They have an important role in maintaining epithelial cell structure, regulating the transport of related ions, and controlling and regulating intestinal permeability (27). The results indicated that LPS could significantly reduce the expression of *Occludin* and *ZO-1* in the intestinal tight junction and increase intestinal permeability. Therefore, there is increased fecal water content and soft and loose stool in LPS mice (26). After the RGO intervention, the expression of *Occludin* and *ZO-1* was significantly enhanced. In combination with the results of intestinal tissue sections, RGO could improve intestinal permeability and water reabsorption capacity. Moreover, it can adjust the balance of water and salt and alleviate diarrhea symptoms by up-regulating the content of intestinal tight junction proteins. Thus, it maintains the integrity of intestinal tissue.

Digestive enzyme activity is one of the essential indicators to evaluate intestinal function, and its primary role is to aid digestion (28, 29). LPS can significantly decrease the activity of pancreatic digestive enzymes in mice. At the same time, the intervention of RGO can substantially improve the activity of pancreatic digestive enzymes, demonstrating that RGO plays a significant role in restoring intestinal dysfunction.

Ninety-five percent of SCFAs in the intestine are acetic acid, propionic acid, and butyric acid, and after being absorbed by the intestine, they store energy and reduce osmotic pressure. SCFAs play an essential role in regulating the normal function of the large intestine and the morphology and function of colonic epithelial cells (30, 31). SCFAs can also enhance the absorption of sodium, especially butyric acid, which can increase the production of *Lactobacillus*. It also reduces the number of *Escherichia coli* and serves as a significant energy source for intestinal mucosal cells (32). This study found that RGO could significantly up-regulate the production of acetic acid, propionic acid, and butyric acid in the intestine. SCFAs can promote the repair of intestinal mucosa damage, restore its function, regulate oxidative stress response, and inhibit the production of inflammatory cytokines as the energy source of intestinal epithelial cells, thus exerting anti-inflammatory effects.

When intestinal inflammation occurs, it can cause an imbalance of intestinal flora. Therefore, it is also crucial to correct intestinal flora and treat intestinal diseases and eliminate inflammation (33). The study observed that endogenous specific anaerobes (Lactobacillus, Bifidobacterium, etc.) could compete against potential aerobic pathogenic bacteria (*Enterococcus faecalis, Escherichia coli*, etc.), and their content could reflect the balance of intestinal flora (34). The results showed that RGO could regulate the intestinal floral structure and restore the balance of intestinal flora in LPS-induced inflammatory mice. This may be facilitated by increasing the relative expression of 16S rRNA genes of intestinal probiotics, including *Firmicutes*, Lactobacillus, and *Akkermansia*. Most *Firmicutes* are beneficial bacteria, such as Lactobacillus, Fecal bacilli, and Lactobacillus. They produce acetic acid and butyric acid in the intestine to enhance the development of intestinal epithelial cells while preventing pathogens from interfering with intestinal health (35, 36). *Bacteroides* can promote the digestion and absorption of lipids, proteins, and carbohydrates. Moreover, they resist the adhesion of invasive intestinal pathogens by colonizing the intestinal mucosa surface (37, 38). Studies have revealed that *Akkermansia* can delay aging, inhibit neurodegenerative diseases, lower lipids, and weight loss, and assist cancer immunotherapy (39). These results indicated that RGO could induce changes in the composition or metabolism of intestinal microbiota, enhance the proliferation of probiotics in intestinal microbiota, and inhibit the growth of harmful bacteria such as *Proteobacteria*. Thus, it affects the structure of intestinal microbiota, regulates the intestinal microecological balance, and maintains the richness and diversity of intestinal microbiota while promoting the health of the host organism. Simultaneously, correlation analysis revealed that beneficial bacteria in the intestine (including *Lactobacillus, Akkermansia*, etc.) were positively associated with intestinal SCFA, tight junction protein, digestive enzyme, and SOD activity. Moreover, they were negatively correlated with the content of inflammatory factors. Thus, RGO may improve intestinal function, reduce inflammation and restore intestinal health by controlling the homeostasis of intestinal flora.

Therefore, RGO can regulate the intestinal floral structure in LPS mice and increase the abundance of intestinal flora. At the same time, it can increase the production of SCFAs in the intestine, decreasing intestinal inflammation, repairing intestinal barrier injury, and maintaining intestinal health. RGO has potential application value in treating and preventing intestinal inflammatory diseases.

## Data availability statement

The original contributions presented in the study are included in the article/supplementary material, further inquiries can be directed to the corresponding authors.

## Ethics statement

The animal study was reviewed and approved by Animal Ethics of the Henan Agricultural University.

## Author contributions

XL and XuebW provided experimental plans and ideas. XL, RG, XuefW, EN, LZ, LC, JZ, ZhL, and YW conducted experiments, sorted out data, and drew drawings. XL, RG, YF, LY, LW, WW, and ZiL helped analyze data and participated in paper writing. All authors have read and agreed to submit the manuscript.
